# Building the Capacity of Adolescents as Researchers: The Co‐Creation of the Health Hive Online Course

**DOI:** 10.1111/hex.70725

**Published:** 2026-06-15

**Authors:** Allyson R. Todd, Sara Wardak, K. Connor, Emma Soo, Elena Wang, Dominik Mautner, Dewa Wardak, Putu Novi Arfirsta Dharmayani, Anna C. Singleton, Josephine Chau, Seema Mihrshahi, Rebecca Raeside, Stephanie R. Partridge, The Health Hive Team, Philayrath Phongsavan, Yvonne Laird, Katrina E. Champion, Lauren A. Gardner, Angela Todd, Hoi Lun Cheng, Karice Hyun, Julie Redfern

**Affiliations:** ^1^ Susan Wakil School of Nursing and Midwifery, Faculty of Medicine and Health The University of Sydney Sydney New South Wales Australia; ^2^ Charles Perkins Centre The University of Sydney Sydney New South Wales Australia; ^3^ Business School The University of Sydney Sydney New South Wales Australia; ^4^ School of Health Sciences and Nursing, Faculty of Medicine, Health, and Human Sciences Macquarie University Sydney New South Wales Australia; ^5^ The Daffodil Centre, a partnership between the University of Sydney, NSW, Australia and Cancer Council NSW Sydney New South Wales Australia; ^6^ The School of Public Health, Faculty of Medicine and Health The University of Sydney Sydney New South Wales Australia; ^7^ Prevention Research Collaboration, School of Public Health, Faculty of Medicine and Health The University of Sydney Sydney New South Wales Australia; ^8^ The Matilda Centre for Research in Mental Health and Substance Use, Faculty of Medicine and Health The University of Sydney Sydney New South Wales Australia; ^9^ Sydney Health Partners The University of Sydney Sydney New South Wales Australia; ^10^ Academic Department of Adolescent Medicine The Children's Hospital at Westmead; ^11^ Specialty of Child and Adolescent Health, Sydney Medical School, Faculty of Medicine and Health The University of Sydney Westmead New South Wales Australia; ^12^ School of Health Sciences, Faculty of Medicine and Health The University of Sydney Sydney New South Wales Australia; ^13^ Institute for Evidence‐Based Healthcare, Bond University Gold Coast Queensland Australia

**Keywords:** adolescent health, co‐design, education, health promotion, participatory action research, youth engagement

## Abstract

**Background:**

Adolescent engagement is critical to ensuring research meets adolescent health and well‐being needs; however, it is not common practice. To maintain research quality, meaningful adolescent engagement requires tailored support and training, yet limited resources exist globally. This study aimed to describe the co‐creation process and outputs of developing a freely accessible online course to build adolescent research capacity in public health (14–24 years).

**Methods:**

An iterative participatory co‐creation research design with four stages, grounded in Positive Youth Development Theory (strengths‐based) and Youth Participatory Action Research (YPAR) principles (participatory, inquiry‐based and transformative). Adolescents, academics and community partners collaborated at every stage as equal knowledge holders. Stage i: An evidence brief was developed synthesising literature reviews and diverse adolescents' lived experience to inform course development. Stage ii: A co‐design workshop was held in March 2025 to design the course structure, style and delivery through interactive group activities, dot‐voting and inclusive dialogue. Stage iii: Content was drafted with iterative rounds of feedback with knowledge holders. Stage iv: Course production and user‐testing, hosted on Open edX.

**Outputs:**

The iterative co‐creation process with 37 knowledge holders (43% 14–24 years) led to an online course structured into six core modules: (1) The power of engaging young people in research, (2) What is public research?, (3) Ethics—how to do research the ‘right way’, (4) Mentally safe participation, (5) How to get involved, and (6) How to apply learnings. Consensus was reached on style and delivery of modules to enhance engagement, content credibility and trust, reflecting adolescent priorities. The course is self‐paced (~3 h duration), strengths‐based, interactive and accessible (e.g., short videos, quizzes, closed captions, audio‐visuals and grade‐8 reading level). It launched in October 2025 and is accessible worldwide.

**Conclusion:**

The co‐created accessible online course aims to support adolescents to be active contributors in public health research. This training has potential to create pathways for inclusive and sustained adolescent engagement in research, translating into evidence‐based research and policy. Effectiveness of the training will be assessed through a mixed‐methods evaluation.

**Patient or Public Contribution:**

Adolescents were involved in all co‐creation stages from inception to delivery, at various capacities within the Health Hive Steering Committee as paid employees. They were provided mentoring and training to fulfil their role throughout each stage. Their contributions included developing the grant proposal, conceptualising the study name and logo, and informing the workshop evidence brief and agenda. They also played an active role at the co‐design workshop, including facilitating and presenting. Further, adolescents informed the course structure, content and delivery of the course and provided iterative feedback during the content creation stage. They also featured in the production of course materials including videos and audio recordings and user‐tested the course. They also engaged as co‐authors of this manuscript (S.W., E.S., K.C., E.W. and D.M.). A checklist adapted by Nagata et al. 2025 of reporting research with adolescent and youth engagement is available in the supporting material.

## 1 Introduction

Adolescence (10–24 years) is a foundational life stage for biological, psychological and neurological development [1, 2]. Supporting the health and well‐being needs of the world's 1.9 billion adolescents is essential for preventing non‐communicable diseases (NCDs) globally and promoting intergenerational equity [3]. However, limited progress has been made in addressing the rising NCD burden [4]. For example, without further intervention, it is projected that a third of children and adolescents and over half of adults globally will be living with overweight or obesity by 2050 [5]. Significant investment, innovation and reorientation in adolescent health research are required [6]. Actively engaging adolescents in NCD prevention research is a crucial first step in developing innovative solutions that meet the health and well‐being needs of adolescents [6].

There are growing calls for adolescents to be active agents of change across public health research and policy [3]. Engaging adolescents in public health research supports a new generation of informed leaders, who will contribute to and shape future health and well‐being outcomes, as well as support countries with an ageing population, such as Australia [3]. Adolescent engagement democratises research, enabling adolescents to contribute to decisions that affect their health and well‐being as well as their communities [7]. In theory, this upholds adolescents' right to participation as outlined in Article 12 of the 1989 United Nations Convention on the Rights of the Child, which was ratified by 189 countries [8]. Meaningfully engaging adolescents involves a mutually respectful partnership with adults, where adolescents feel valued, enabling them to contribute and share their ideas, perspectives, skills and strengths to shape decisions impacting their lives and communities [9].

Meaningful adolescent engagement in public health research provides multilevel benefits [10]. On an individual level, it builds adolescent research capacity, defined as the foundational knowledge and skills required to actively engage in research beyond being the participants of research [11]. When mental well‐being safeguards are implemented, participatory research can increase adolescent resilience, agency, and leadership skills [10, 12, 13]. Further, when adolescents are empowered to pursue their interests and learning, it leads to a greater sense of purpose and contributions to civic society, which is central to well‐being [12, 13, 14]. It also prepares the future workforce, supporting career pathways in health, science and research. There are positive benefits for the researchers involved as well as improved research processes and outcomes [10]. This includes improved research relevance, study recruitment and retention, and dissemination of findings [15, 16]. Ultimately, this can lead to improved policies and programs that reflect adolescent needs and greater health and well‐being outcomes [10]. Due to these wide‐ranging benefits, there is growing momentum globally to engage adolescents across organisations and governments at various levels. This extends to academia, including granting bodies and peer review journals commonly requesting adolescent or consumer involvement statements [10].

Despite growing demand to engage adolescents in public health research, multiple barriers exist. Within the last 5 years, seven major reviews report a lack of resources, training and support to build adolescent research capacity [17, 18, 19, 20, 21, 22, 23]. An umbrella review of 99 reviews, encompassing 2926 individual research studies, identified few mitigation strategies implemented to address these barriers [17]. Building adolescent research capacity is necessary to maintain research quality, as well as reduce tokenistic involvement [22]. A qualitative study examining youth and adult perspectives highlighted that good preparation and planning, accounting for capacity building, is required to ensure meaningful adolescent engagement [24]. Further, consultations with over 500 adolescents living in Australia revealed demand for greater support to gain skills and leadership, ensuring adolescents feel confident to meaningfully contribute across the research cycle in NCD prevention [25]. This is similarly reported in the United Kingdom, where adolescents identified academic jargon and a lack of understanding of complex research processes as a barrier to engaging in health research [26]. This highlights the need for accessible pathways to build adolescent research capacity.

Current training approaches to build adolescent research capacity are limited by accessibility, reach and scalability. An environmental scan of empirical studies and grey literature in 2019 found 13 training programs available to increase adolescent engagement in health policy and advocacy [27]. Most programs were limited to in‐person, with long durations, and lacked reporting of how they were created and if adolescents were involved [28]. Additionally, most adolescent engagement frameworks, guidelines and resources are context‐specific or siloed to specific health conditions, with limited adolescent input, and minimal focus on NCD prevention [14, 29, 30, 31]. The most common strengths‐based approaches to build adolescent capacity are Positive Youth Development Theory and Youth Participatory Action Research (YPAR) [29, 32]. Adolescents are not a homogeneous group; therefore, it is important to create universally accessible training resources with adolescents to enhance usability and acceptability [33].

Within a rapidly digitised world, adolescents are seeking formal and informal education pathways online to gain health knowledge and skills [33]. Online courses have the potential to provide accessible and scalable pathways to build adolescent research capacity [34]. A rapid review found 23 free online courses for adolescents, yet none focused on upskilling in public health research [34]. The review found effective strategies that support adolescent learning needs, including non‐lecture videos, quizzes and reading materials. Additionally, the review identified enablers influencing learner engagement. This included personal benefit, interest, sense of accomplishment, user satisfaction, interactivity, credibility of information, and ease of language used. Further, adolescent‐centred, health‐promoting universal online courses hold promise in increasing adolescent knowledge and skills when adolescents are meaningfully involved [34]. However, detailed reporting of co‑creation processes and adolescents' active involvement in public health research remains limited in the literature [1, 35]. Therefore, this study aims to describe the co‐creation process and outputs of developing a freely accessible online course (herein referred to as the Health Hive online course) to build adolescent capacity in public health research.

## 2 Methods

### 2.1 Study Design

**Figure 1:**
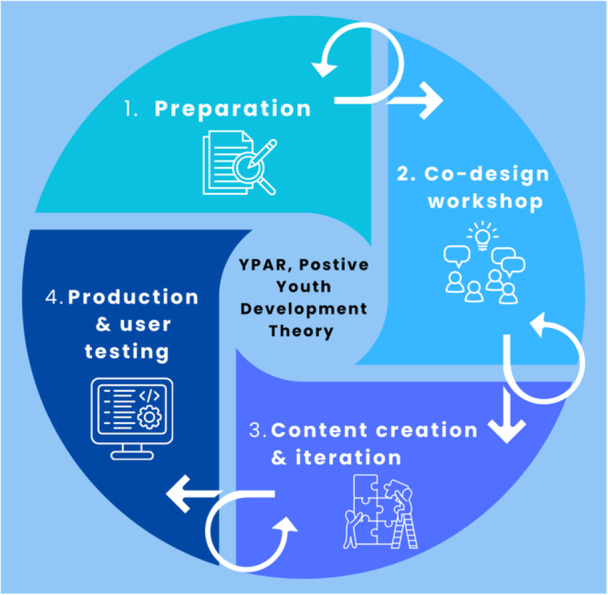
Co‐creation Stages. Post implementation, the course continues to be improved through ongoing monitoring, evaluation and incorporation of learner feedback. YPAR = Youth Participatory Action Research.

We used an iterative approach to co‐create the Health Hive online course. Co‐creation is defined as the overarching process of actively involving key knowledge holders from inception to implementation of solutions who provide continuous input [36, 37]. In this study, knowledge holders refer to individuals with relevant professional or lived experience expertise. Co‐design is an element of co‐creation which specifically focuses on prioritising knowledge holders' needs, expertise and knowledge to develop solutions [35]. In this study, we conducted four co‐creation stages over 1.5 years: (i) preparation, (ii) co‐design workshop, (iii) content creation and iteration, (iv) production and user testing (Figure 1).

### 2.2 Theoretical Approaches

The co‐creation process was informed by Positive Youth Development Theory [29]. This strengths‐based approach positions adolescents as active agents of change to make a positive difference in their communities. Further, it promotes opportunities for growth, enabling adolescents to thrive and reach their potential. A YPAR approach was also applied to establish an equal partnership between adolescent and adult researchers whilst building adolescent team members' research capacity [38]. YPAR is a participatory, inquiry‐based and transformative approach that empowers adolescents to meaningfully contribute to research and shape positive health outcomes [39].

### 2.3 Context and Setting

This study is part of a wider co‐creation project which includes four main components: (1) the Health Hive online course for adolescents to upskill in public health research and advocacy; (2) Community of Learners shaped by adolescents who take part in the Health Hive online course; (3) Opportunities Hub to make pathways into public health more accessible and visible to adolescents, connecting them with diverse ways to contribute including youth advisory groups [40]; (4) Partnerships for Impact which aims to amplify adolescent engagement in the broader public health research community. The Health Hive online course was developed within an Australian setting with an NCD prevention lens to support research aimed at improving adolescent health and well‐being. This study was supported by an Australian Government Medical Research Future Fund (MRFF) Consumer‐Led Grant (#2023165), which provided funding for adolescent salaries, resources to co‐create the Health Hive course across each stage, and course hosting.

### 2.4 Study Team and Roles

In total, 37 knowledge holders were involved in the co‐creation of the Health Hive online course, with 43% (*n* = 16) aged 14–24. Most knowledge holders were part of the Health Hive Steering Committee (*n* = 26) (herein referred to as the Steering Committee), which led this study. The Steering Committee comprises Chief and Associate Investigators of an MRFF Consumer‐Led Grant (#2023165), project staff, community partners (Sydney Health Partners, the Australian Cardiovascular Alliance, and Health Consumers NSW), and adolescents. Collectively, adult knowledge holders brought expertise in adolescent engagement, citizen science, public health, health promotion, digital health, education design and implementation science. Further, eight adolescent Steering Committee members were paid employees at the University of Sydney (job title ‘Peer Facilitators,’ *n* = 5, and ‘Youth Research Assistants’, *n* = 3), seven of whom were in high school upon commencing their role. They all have lived experience and expertise in engaging in public health research as youth advisors and co‐researchers. Additional adolescent knowledge holders involved include master's students and high school youth advisors from the 2024 Health Advisory Panel for Youth at the University of Sydney (HAPYUS) cohort.

### 2.5 The Co‐Creation Process

#### 2.5.1 Stage i: Preparation

Peer Facilitators were recruited to co‐create a free online course in May 2024. To accommodate school and university study schedules, flexible and remote working arrangements were in place. This also supported Peer Facilitators' accessibility needs, including those living in non‑metropolitan areas. In‐person workshops were held during school and university holidays. Digital devices (including laptops and mobile phones) and internet access packages were provided if needed, ensuring digital equity [41].

Peer Facilitators were tasked to regularly reflect and write about their lived experiences of being involved in adolescent engagement initiatives, as well as their experiences joining the research team. They were prompted to reflect on what training they wish was available at the time to help them feel confident in their new role or when first taking part in public health and what would help their peers get involved in research [42]. Youth advisors from HAPYUS were also consulted via Zoom to share their reflections to inform the course development. Additionally, Peer Facilitators developed the course identity, including the project name and logo.

An evidence brief was developed by ART to inform the co‐creation of the Health Hive online course. It contained an evidence synthesis of global literature, which is also summarised in the introduction, and insights from adolescent lived experiences. The evidence brief was circulated to knowledge holders prior to attending the co‐design workshop.

#### 2.5.2 Stage ii: Co‐Design Workshop

Twenty‐one knowledge holders attended a 1‐day in‐person co‐design workshop in March 2025. Just over a third of attendees (*n* = 8, 38%) were adolescents aged under 24 years. The aim of the workshop was to reach consensus on the course structure, core content topics, delivery and style. The co‐design workshop was led and facilitated by A.R.T. and S.W. through an Appreciative Inquiry approach [43, 44]. It focused on strengths, sharing positive experiences, and imagining the best learning experience. Living lab methodology was utilised, which is a collaborative and adaptive approach that integrates diverse perspectives to develop solutions [45]. Inclusive dialogue ensured a supportive environment [33, 46]. The workshop began with icebreakers followed by youth engagement reflections (S.W. and D.M.), ensuring all workshop outputs were tailored for adolescent end users.

The workshop was structured through dream, design and deliver sessions (see Supporting Information). Each session involved breaking into smaller groups, with representation from at least one adolescent in each group. The groups brainstormed and documented their ideas on butcher's paper. At the end of each session, each group presented back to the wider group, inviting further discussion and reflections. Briefly, in the dream stage, attendees imagined what an ideal course would look like, including ways to make the course engaging, practical and informative. In the design stage, module topics were brainstormed. To narrow down core module topics to include in the Health Hive online course, dot voting was used. This is a common group decision‐making method using stickers or tokens to quickly capture group preferences [47]. Module topics with the majority (> 50%) votes were prioritised while ensuring all adolescent knowledge holders agreed. Next, learning objectives were mapped out for each module. In the deliver stage, the course structure, delivery and style were mapped out and finalised with consensus reached. Photographs were taken of all notes made at the end of the workshop. A graphic illustrator attended the workshop to capture visual summaries of each session and the evolution of ideas.

#### 2.5.3 Stage iii: Content Creation and Iteration

Following the co‐design workshop, course content was developed with iterative rounds of feedback and refinement with all knowledge holders in the study team. All content was drafted based on decisions made at the co‐design workshop. The content developed, including topics to build adolescent research capacity, was evidence‐based, informed by the literature and collective expertise of the Steering Committee. Each module was drafted, outlining the intended structure, content and learner experience envisioned for course production. The Sydney Health Literacy editor tool (SHeLL Editor) was used to ensure accessible language was embedded throughout the course, including video and audio scripts, aiming for a reading level of grade 8 or lower [48, 49]. Course videos were filmed in July 2025, during school and university holidays by a professional videographer. Audio clips and short video reels were recorded by Peer Facilitators via iPhone 16 Pro and a mini microphone.

#### 2.5.4 Stage iv: Production and User Testing

The Health Hive online course was developed using Open edX, which is an open‐source learning management system. It enables a large‐scale, accessible learning environment and has supported over 100 million learners globally [50]. Aulasneo were contracted to host the platform on Open edX and provide technical support [51]. They provide digital infrastructure which complies with the Amazon Web Services (AWS) Well‐Architected Framework, which includes pillars such as Security, Reliability and Operational Excellence, enabling best practice design and delivery of scalable online courses [52].

The Health Hive online course was user tested by five adolescent knowledge holders prior to public launch. They independently completed the course and provided feedback to improve course navigation, usability and engagement, which was incorporated into the final version. They also identified any errors, including expired web‐links or technical issues, which were resolved prior to launching the course. The Patient Education Materials Assessment Tool (PEMAT) was used to review the course's accessibility. It is an instrument used to improve the understandability and actionability of audiovisual education materials [53].

## 3 Outputs of Co‐Creation

### 3.1 Course Identity

The Peer Facilitators created the project name and logo, which led to the formation of the ‘Health Hive’ and slogan ‘Together We Thrive’. It is inspired by connection and collaboration to create a thriving community to shape healthier futures.

### 3.2 Course Structure and Learning Objectives

**Figure 2:**
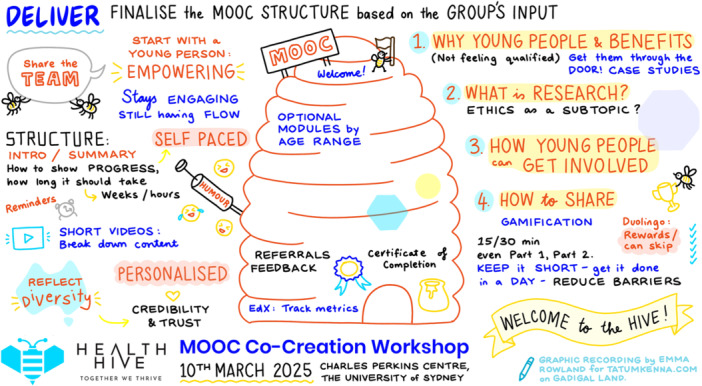
Graphic illustration of the co‐design workshop.

**Figure 3:**
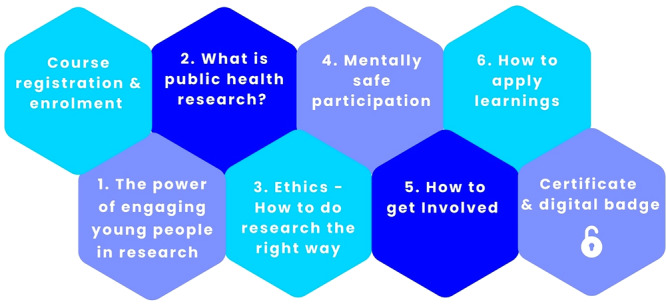
Course structure.

Figure 2 illustrates the consensus reached during the co‐design workshop informing the outputs of this study. All graphic illustrations from the workshop are available in the Supporting Information. The Health Hive online course structure is outlined in Figure 3 and comprises six core modules: (1) The power of engaging young people in research, (2) What is public health research? (3) Ethics—how to do research the ‘right’ way, (4) Mentally safe participation, (5) How to get involved, and (6) How to apply learnings. Upon completion, learners can download a certificate and digital badge.

A summary of course learning objectives is provided in Table 1. Course content draws from global evidence and practical case studies to provide a high‐level overview of how adolescents can actively get involved in public health, including research, advocacy and community projects through consultation, collaboration and adolescent‐led approaches [55].

**Table 1:** Health hive module learning objectives.

Module	By the end of each module, learners will understand the following
1. The power of engaging young people in research	The importance of involving young people in research that affects them How to debunk common myths about research and young people's roles The benefits gained from being involved in research How to ensure they have a good experience when being involved in research, and what to do if things do not seem right
2. What is public health research?	Part 1 What is public health The benefits of public health research Types of public health challenges How public health research is used and applied in everyday life Who is involved in public health research, and where does it happen Part 2 The types of evidence used in public health research Common study designs involving adolescents (e.g., but not limited to randomised controlled trials, longitudinal and cohort studies, qualitative and mixed‐methods research, and systematic reviews). Common research methods The different stages of research
3. Ethics—How to do research the ‘right way’	What is ethics Why ethics is needed in research How to be an ethical researcher
4. Mentally safe participation^a^	Organisations have a duty of care to keep young people safe Why it is important to make sure young people feel mentally safe when they take part in projects Guidelines to help protect young people's mental health and well‐being Where to go for help and support Tips for looking after yourself (self‐care)
5. How to get involved	Different ways to take part in public health, including research, advocacy and community action projects (e.g., a youth advisor, co‐researcher or advocate). Tips on getting started in a new role and how to manage expectations How to find opportunities
6. How to apply learnings	Tips to help young people succeed in public health research and advocacy How to turn their ideas into actions to improve community health How to seek out opportunities or lead their own project

^a^ Module 4 was informed by the Mentally Safe Participation guidelines by Guo et al. (2024) [54] and a scoping review led by Donohoe‐Bales et al. (2025) [12].

### 3.3 Delivery, Engagement and Accessibility Features

The Health Hive online course is delivered online with no live interactions, enabling learners the flexibility and autonomy to complete the course at their own pace. Whilst a recommended structure is provided, learners may navigate modules non‑linearly and prioritise topics of interest. Each module takes on average 30 min to complete. It takes approximately 3 h to complete the course entirely. The course is tailored for adolescents 14–24 years, with no prior background knowledge or experience required. It is a legal requirement for edX learners to be aged 13 years or above [56].

To maximise engagement, it was unanimously decided that all videos should be short and concise, in a non‐traditional lecture style. Each module contains a variety of professionally filmed and short reel videos. Videos feature a mix of adolescents and academics to enhance content credibility and trust. Video length ranges from 20 s to 1 min 54 s, with an average length of 1 min (Table 2).

**Table 2:** Summary of course content.

	Frequency (*n*)	Duration range	Mean duration per item
Professional videos	36	35 s to 1 min 54 s	1 min 7 s
Short reel videos	10	20 s to 1 min 8 s	41 s
Total videos	46	20 s to 1 min 54 s	1 min
Videos featuring adolescents, *n* (%)	26 (56.5%)	—	—
Audio files	37	16 s to 1 min 6 s	34 s
Interactive activities^	43	—	—
Downloadable templates, guides and module summaries	14	—	—

*s = seconds, min = minute. ^includes multiple choice quizzes, polls, drag and drop tasks, open text reflections, and pop‐up information tiles.

Additional engagement strategies included embedding gamification elements within the course. Auditory and digital rewards were included to establish a sense of achievement when completing a module and the course, including a certificate and digital badge. Further, learners can track their progress on the side panel, which appears green once complete. Each module includes interactive activities or multiple‐choice quizzes with immediate feedback.

Accessibility was a key consideration. The course caters to different learning styles by offering a mix of audio, visuals and text in each module. All videos include closed captions. Further, all written text has accompanying audio recordings. Each module includes a downloadable summary. Guides and templates are also provided to help users implement learnings, such as how to seek out a mentor and tips for applying for community grants to fund youth‐led projects. All content has a reading score of 8 or lower [49]. If complex terms could not be simplified, they were included in a glossary list.

### 3.4 Implementation Milestone

The Health Hive online course “Foundations in Public Health Research & Advocacy for Young People” was made publicly available on 30 October 2025. The course launched at the Australian Association for Adolescent Health (AAAH) Youth Health Showcase at the Parliament House of Australia, in front of federal politicians, policy advisors, researchers and young people. The course is freely accessible worldwide. Learners can self‐register via the Health Hive website [57]. The course can be completed on any computing device, including a laptop or smartphone. As of 12 June 2026, 827 people have enrolled from 47 countries.

## 4 Discussion

### 4.1 Principal Output

Our study describes the process of co‐creating Australia's first freely accessible online course to build adolescent research capacity. Whilst the Health Hive online course was developed in an Australian context, it is freely accessible worldwide. Through an iterative co‐creation process, six core modules were developed to provide foundational training. The course is strengths‐based and grounded in Positive Youth Development Theory to build adolescent knowledge, skills and confidence to meaningfully contribute to public health research. Drawing from global evidence, the course provides practical case studies and templates supporting adolescents to get involved and shape healthier futures. The Health Hive online course aims to address a significant gap in the literature, evident by seven major reviews reporting a lack of resources, training and support to build adolescent research capacity in public health [17, 18, 19, 20, 21, 22, 23].

### 4.2 Comparison to the Literature

The co‐created module topics within the Health Hive online course are consistent with other initiatives aiming to improve adolescent research capacity. The MyHealth study is currently testing the efficacy of a virtual training program for adolescents living in Michigan, the United States [58]. The program is focused on building knowledge and skills in research, including ethics, data collection, data analysis and dissemination, as well as promoting science, technology, engineering and mathematics (STEM) careers [16]. Similarly, other researchers in Michigan have developed an in‐person school curriculum for older high school students to upskill the future public health workforce, where they can earn college credit at Michigan University [59]. The curriculum covers similar content topics offered by the Health Hive online course, including introduction to public health, importance of research, common research methods and complemented by case studies. It has now been embedded across nine Michigan high schools and has been adapted across six countries, including India, Saudi Arabia, Turkey and Portugal [59]. Similarly, LifeLab has been implemented within selected high schools in Sydney, Australia, to educate students about health through a scientific lens, encouraging STEM career pathways [60]. However, these initiatives are currently limited to either in‐person attendance or living within a specific location. The Health Hive online course offers a scalable reach for adolescents to build capacity in public health research regardless of geographical location.

The digital determinants of health, including access, affordability, ease of use, interactivity and digital literacy, were considered in the co‐creation process of the Health Hive online course to improve digital equity [41]. Research has shown that failure to consider these factors when developing digital interventions may inadvertently widen the digital divide and reinforce health disparities amongst adolescents [61, 62]. We aimed to address these determinants throughout each co‐creation stage. For example, Peer Facilitators were supported to work remotely with adequate internet and digital devices. Iterative rounds of user testing with adolescents also improved the ease of navigation and interactivity with the course. Further, a variety of methods were utilised to increase the accessibility of the course. This includes the use of closed captions, short videos, complementary audio recordings, a recommended grade reading level of all text, a glossary index and module summaries available to download. These accessible co‐design outputs have similarly been reported in Canada by youth aged 18–25 years living with neurodevelopment disabilities wanting to upskill in research [63]. The Health Hive online course was also made freely available to the public, which can be completed on any computing device, including a smartphone. Within Australia, it is estimated that 96% of adolescents aged 14 or over have access to a smartphone [64]. Whilst most adolescents living within high‐income countries have access to the internet and a digital device, it is estimated that only one‐third of adolescents have internet access in low‐income countries [65]. Future iterations will aim to improve acceptability for low‐resource settings and different regional contexts.

### 4.3 Practical Implications for Research, Policy and Institutions

Evident through our co‐creation process, adequate resources are needed to execute meaningful adolescent engagement in public health research [19, 66]. Significant time was invested in mentoring and supporting Peer Facilitators to navigate a traditionally adult‐oriented workplace, including human resources onboarding processes. Flexibility was also crucial to ensure Peer Facilitators could meaningfully contribute, with team meetings often scheduled outside traditional work hours to accommodate adolescents' school schedules. Sustaining adolescent engagement through multi‐year projects can be challenging [17]. Throughout the study, two Peer Facilitators resigned to pursue their career interests after completing high school. Being adaptive in our project management enabled us to successfully launch the Health Hive online course to the public. Additionally, implementing a YPAR approach helped mitigate power dynamics and balance diverse perspectives throughout the co‐creation process. The Steering Committee were committed to creating a supportive environment where everyone's ideas were valued, supported by a participatory governance structure. Consistent reflection, feedback loops and transparency ensured each knowledge holder's input felt valued and considered [67]. Additional mitigation strategies included anonymous feedback mechanisms, such as post‑workshop surveys to support open dialogue, alongside youth‐led facilitation across each stage, ensuring adolescent voices were prioritised. As reflected by the Peer Facilitators, they experienced genuine power sharing and were integral contributors in shaping the Health Hive online course [68].

Whilst building adolescent capacity is important to maintain research quality, it is important to recognise that the onus should not be on adolescents themselves [24]. Researchers have an obligation to ensure adolescents engage in a safe and supportive environment [54]. Further, researchers need to meet adolescents where they are at, recognise their strengths, and provide appropriate training to fulfil their roles [28]. To address this, researchers need to appropriately plan for resources when budgeting for projects to ensure meaningful adolescent engagement in public health research [24]. As voiced by adolescents, investing in adolescent capacity building is particularly important when researchers have limited funding and are unable to provide financial compensation or remuneration [24]. The Health Hive online course aims to deliver accessible support for both adolescents, researchers and organisations who work with young people to build public health research capacity.

Reporting on the process of co‐creating the Health Hive online course is an important step in increasing the transparency and applicability of best practices of meaningfully engaging adolescents in public health research [66, 69]. There are ongoing calls for researchers to implement minimum standards for co‐design, with clearly defined principles [33, 70], and standardised reporting of the level of adolescent involvement in health research [1]. Further, there is a need for robust evaluations assessing the impact of adolescent engagement in public health research [10]. A pre‐ and post‐course survey has been embedded within the Health Hive online course to inform a mixed‐methods evaluation. Assessing the implementation and application of the Health Hive online course will enable future iterations that meet adolescents' needs.

### 4.4 Strengths and Limitations

A key strength of this study is the co‐creation methodology, which promoted the active involvement of adolescents across all co‐creation stages, including the grant proposal. Adolescent engagement in research often occurs after funding is received with a pre‐planned protocol, restricting authentic co‐creation [37]. Our collaborative, iterative and generative approach provided agency to adolescent knowledge holders to shape the design and delivery of the Health Hive online course [35]. This is supported by the Peer Facilitators' reflections on the co‐creation process, which are reported elsewhere [68]. Another key strength is that the Health Hive online course addresses a significant gap in the literature. The course provides free, scalable infrastructure, supporting researchers and organisations to build adolescent research capacity in public health. It also responds to demand from adolescents worldwide for accessible training and support to get involved in shaping healthier futures [24, 25, 71].

Whilst we engaged a diverse team of adolescents in the co‐creation of the Health Hive online course, it is not representative of all adolescents. Learner feedback will improve future iterations of the course. Further, the course was funded to focus on NCD prevention. Whilst the Health Hive online course provides accessible teachings and transferable skills, it does not offer context‐specific training for people with a particular lived experience of a health condition or disease. This highlights an opportunity for future research, where adolescents may require more tailored support, training and onboarding. Further, knowledge holders engaged as part of the research team, which reduced power imbalances. However, this limited our ability to independently evaluate the co‐creation process, which was beyond the scope of this study. Additionally, as we were directly involved in co‐creating the Health Hive online course, we acknowledge this may bring potential bias in reporting of how we meaningfully engaged adolescents. To mitigate this, we aimed to report the co‐creation process as transparently as possible, with direct input from adolescent co‐authors [72, 73]. Whilst substantial efforts were made to make the course accessible and scalable, it is currently restricted to the English language and learners who have access to the internet and a digital device. Additionally, cultural adaptation will be required to enhance the local relevance of the course across regions. Further, the sustainability of offering this freely accessible online course will depend on securing ongoing funding.

## 5 Conclusion

This study describes the co‑creation process and outputs of developing the Health Hive online course with adolescents. The iterative participatory process with diverse knowledge holders enabled us to respond to adolescents' needs and priorities by developing a freely accessible online course to build adolescent research capacity in public health. The Health Hive online course aims to provide an accessible infrastructure for researchers to increase meaningful adolescent involvement in public health research. It is envisioned that the Health Hive will foster transferable skills and connect adolescents to ongoing opportunities to shape healthier futures and reduce the NCD burden.

## Author Contributions

**Allyson R. Todd:** conceptualisation, investigation, writing – original draft, writing – review and editing, methodology, project administration, visualisation, formal analysis. **Sara Wardak:** writing – review and editing, investigation, methodology. **K. Connor:** writing – review and editing, investigation, methodology. **Emma Soo:** writing – review and editing, investigation, methodology. **Elena Wang:** writing – review and editing, investigation, methodology. **Dominik Mautner:** writing – review and editing, investigation, methodology. **Dewa Wardak:** writing – review and editing, investigation, methodology. **Putu Novi Arfirsta Dharmayani:** writing – review and editing, investigation, methodology. **Anna C. Singleton:** writing – review and editing, investigation, methodology. **Josephine Chau:** writing – review and editing, investigation, methodology. **Seema Mihrshahi:** writing – review and editing, supervision, investigation, methodology. **Rebecca Raeside:** writing – review and editing, supervision, investigation, methodology. **Stephanie R. Partridge:** writing – review and editing, supervision, funding acquisition, conceptualisation, investigation, methodology. **Philayrath Phongsavan:** writing – review and editing, investigation, methodology. **Yvonne Laird:** writing – review and editing, investigation, methodology. **Katrina E. Champion:** writing – review and editing, investigation, methodology. **Lauren A. Gardner:** writing – review and editing, investigation, methodology. **Angela Todd:** writing – review and editing, investigation, methodology. **Hoi Lun Cheng:** writing – review and editing, investigation, methodology. **Karice Hyun:** writing – review and editing, investigation, methodology. **Julie Redfern:** writing – review and editing, investigation, methodology.

## Ethics Statement

Ethics approval was not required as adolescents were engaged as members of the research team (not study participants) and were co‐authors of this article. No personal health or demographic data were collected or reported. This approach was supported by advice from the University of Sydney Human Research Ethics Committee and aligns with the Australian National Health and Medical Research Council Statement on Consumer and Community Involvement in Health and Medical Research.

## Conflicts of Interest

The authors declare no conflicts of interest.

## Supporting information

Supporting File 1

Supporting File 2

**Table S1:** Checklist for reporting research with adolescent and youth engagement.

## Data Availability

Data sharing is not applicable to this article as no datasets were generated or analysed during the current study.
