# Supplementary material for: Building the Capacity of Adolescents as Researchers: The Co‐Creation of the Health Hive Online Course
Source: Health Expect. 2026 Jun 15;29(3):e70725. doi: 10.1111/hex.70725 (PMC13269657; doi:10.1111/hex.70725)
Supplement: Supplementary file 1 — Supporting File 1 [file HEX-29-e70725-s003.pdf]

ONLINE COURSE CO-DESIGN WORKSHOP

Date & Time: Monday 10th March, 9am – 3pm

Location: Mackenzie Room, Level 6, Charles Perkins Centre, The University of Sydney, Camperdown 2050

AGENDA

1. Acknowledgement of country

2. Introduction (9 - 9:30am)

- Housekeeping
- Icebreaker
- Workshop objective: Co-design the structure, delivery and style of the course
- Method: Appreciative inquiry (focusing on strengths, sharing positive experiences, and imagining the best learning experiences) incorporating a Youth Participatory Action Research Approach
- Youth engagement reflections & Q/A

3. Appreciative inquiry & dream (9:30 - 10am)

- Purpose: identify key positive learning experiences that will inform the course design and content
- Activity: Form groups of 4-5 (each group with at least one young person)
  - Discuss your reflections to the following questions (and take notes on writing pad)
    - (a) What's the best online course or learning experience you've had, and why was it so effective? What made it enjoyable?
    - (b) If you could design the perfect online course, what would it look like? What kinds of activities, content and structure would make you excited to learn?
  - Share your group's insights with the whole group

MORNING TEA (10 – 10:30am)

4. Mapping content sections (10:30 – 11am)

- Purpose: brainstorm and prioritise content for modules
- Activity: In groups, write down content topics for modules
  - Use a quick poll (dot-voting) to prioritise the most important sections or content types (these are must-haves)
  - Identify which topics should be core content modules or an elective

5. Design (11 am – 1pm)

- Purpose: Design the content and structure of the course modules
- Activity: In groups, write down content topics for modules
  - Define the section's purpose and goal i.e. Learning objectives for that module
  - Identify key topics to include in the section
  - Decide content types. E.g., Videos, readings, hands-on activities, case studies etc.
  - Sketch/map out the rough structure of the section. E.g., video, quiz, discussion
  - Think about how to make it engaging. E.g., Multimedia, peer collaboration, polls etc.
- 12pm: Each group presents their section in 5 minutes, explaining what it includes and why it is important
- 12:50pm: Questions
- Group photo for website

LUNCH (1 – 2pm)

6. Youth engagement reflections & Q/A (1-15pm)

7. Deliver (2:15pm– 2:45pm)

- Purpose: Finalise the course structure based on the group's input
- Activity: Add thoughts/ideas to other groups notes to see if anything was missed
- Group discussion and refinement
  - Create a visual final display of the outline of the course sections and the content that will go in each

8. Closing (2:45 – 3pm)

- Reflections
- Next steps after the workshop
- Questions?
