# Supplementary material for: Building the Capacity of Adolescents as Researchers: The Co‐Creation of the Health Hive Online Course
Source: Health Expect. 2026 Jun 15;29(3):e70725. doi: 10.1111/hex.70725 (PMC13269657; doi:10.1111/hex.70725)
Supplement: Supplementary file 2 — Supporting File 2 [file HEX-29-e70725-s001.pdf]

INTRODUCTION

WORKSHOP OBJECTIVE: CO-CREATE the STRUCTURE of the MOOC

ACKNOWLEDGEMENT of COUNTRY

The GADIGAL PEOPLE of the EORA NATION

Welcome!

ICEBREAKER

Guess who!?

Meeting your TEAM

'The CLOUD' Questions

"BLUE SKY THINKING"

HAVE FUN!

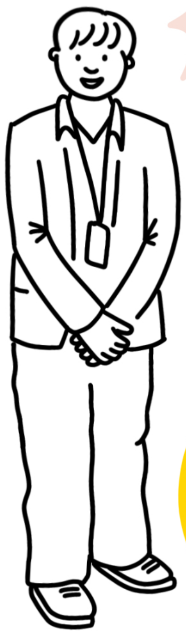

WORKING with YOUNG PEOPLE

- Explain EVERYTHING in detail
- Be PATIENT
- ACCESSIBILITY: Connect with ALL Young People

APPRECIATIVE INQUIRY

EMERGED in the 1980's

with DEWA

- FOCUSING on STRENGTHS
- SHARING POSITIVE EXPERIENCES
- IMAGINING the BEST LEARNING EXPERIENCES

DISCOVER
DREAM
DESIGN
DELIVER

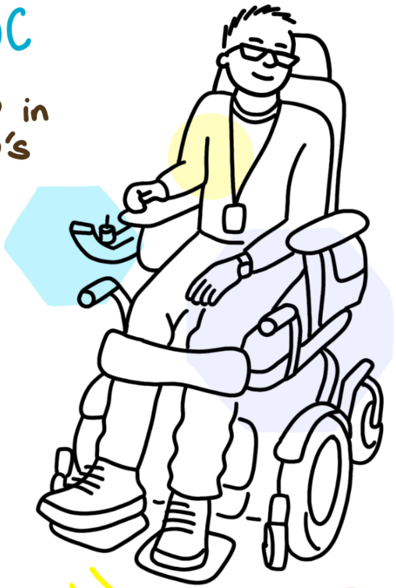

YOUTH PARTICIPATORY ACTION RESEARCH APPROACH

- ✓ INQUIRY based
- ✓ PARTICIPATORY
- ✓ TRANSFORMATIONAL

Collaborative

YOUTH ENGAGEMENT REFLECTIONS & Q/A

"People are EXPERTS in their OWN LIVED EXPERIENCE"

with SARA

First experience: Year 10 Westmead WORKSHOP

That would be fun!

Articles 3, 12, 24

There are AMAZING BENEFITS!

WHY is CO-CREATION IMPORTANT?

- "WORK for WHO it SHOULD WORK for"
- HUMAN RIGHTS
- DEMOCRATISING

HEALTH HIVE
TOGETHER WE THRIVE

MOOC Co-Creation Workshop

10TH MARCH 2025 CHARLES PERKINS CENTRE, THE UNIVERSITY of SYDNEY

GRAPHIC RECORDING by EMMA ROWLAND for TATUMKENNA.COM on GADIGAL LAND.

APPRECIATIVE INQUIRY & DREAM

IDENTIFY KEY POSITIVE LEARNING EXPERIENCES THAT WILL INFORM THE MOOC'S DESIGN & CONTENT

WHAT'S the BEST ONLINE COURSE or LEARNING EXPERIENCE YOU'VE had & WHY? What made it ENJOYABLE?

MOTIVATION: "SELL the COURSE"

RELEVANCE

Look forward to

"TOPIC of the WEEK"

"CAMERA ON"

Engaging

ICE BREAKERS

MATCH STUDENTS & tutors based on AGE / INTERESTS

INTERACTION

RECIPE VIDEO style

BRIGHT COLOURS

DARK MODE

CC

Alt text

ACCESSIBILITY

LANGUAGES

Break out ROOMS

SAME experience as IN PERSON

Calling on PEOPLE, giving FEEDBACK

Self assess / QUIZZES

With a young child at home

PODCASTS

MUST DO / Optional TASKS

LEARNING to your STRENGTHS

Creativity

FLEXIBLE

- Spanish
- Coursera
- Duolingo

MONITORED

Cater to different LEARNING STYLES

A REAL PERSON (More personal)

Interview STYLE VIDEOS

Progress Bar

WHAT WOULD your PERFECT ONLINE COURSE LOOK LIKE? WHAT would make you EXCITED to LEARN?

LIVE

MAKING VIDEOS

- ACTIVITIES
- CONTENT
- STRUCTURE

Being able to do EXPERIMENTS

TASK oriented

Bite sized

DOWNLOADABLE SUMMARIES

Be able to come back to it

SOCIAL DISCUSSION BOARD

Self PACED

- Q & A
- Glossary
- Abbreviations

HEALTH HIVE
TOGETHER WE THRIVE

MOOC Co-Creation Workshop

10TH MARCH 2025

CHARLES PERKINS CENTRE, THE UNIVERSITY of SYDNEY

GRAPHIC RECORDING by EMMA ROWLAND for TATUMKENNA.COM on GADIGAL LAND.

MAPPING CONTENT SECTIONS

BRAINSTORM & PRIORITISE CONTENT for MODULES

ADOLESCENTS as CO-RESEARCHERS

STEREOTYPES

WHAT is RESEARCH?

WHAT is a MOOC?

"What's a PERSPECTIVE?"

"What is a LITERATURE REVIEW"

Difference between LABORATORY & FIELD WORK

e.g.
- Reviewing DOCUMENTS
- Acting as an ADVISOR
- Co-Authoring PUBLICATIONS

TYPES of RESEARCH / PROCESSES

OUTPUTS

HOW to read a PAPER

EMPOWERMENT /AGENCY

ETHICS

HOW to EXPRESS your EXPERIENCE?

WHAT are they LOOKING for?

COMMUNICATION

MODES: PARTICIPANT VS. CO-CREATION

ROLES & RESPONSIBILITIES

WHICH TOPICS SHOULD be CORE CONTENT MODULES or an ELECTIVE?

What does INVOLVEMENT look like?

CONTRIBUTING
- Focus groups
- Surveys

QUALIFICATION? Young & passionate

VALUABLE!

LIVED EXPERIENCE

HOW to GET INVOLVED?

- MOTIVATIONS
- BENEFITS

e.g. TRANSFERRABLE SKILLS
- Research
- Critical thinking
- Understanding Evidence

YAG MAP

interactive

CASE STUDIES

EXAMPLES

What's in it for YOU?

EVIDENCE

What is HEALTH?

LENS

Different ways of SHARING KNOWLEDGE

CORE LEARNINGS
* Climate change
* Social Justice

A PLACE for STUDENTS to CONNECT

END of MOOC: Community of Practice

IMPLEMENTING your LEARNINGS: HOW to USE in COMMUNITY

HEALTH HIVE

TOGETHER WE THRIVE

MOOC Co-Creation Workshop

10TH MARCH 2025

CHARLES PERKINS CENTRE, THE UNIVERSITY of SYDNEY

GRAPHIC RECORDING by EMMA ROWLAND for TATUMKENNA.COM on GADIGAL LAND.

DESIGN

DESIGN the STRUCTURE of the MOOC MODULES CORE TOPICS

WHAT is RESEARCH?

DEBUNK STEREOTYPES

Methodology: QUAL. vs QUANT.

DISCUSSION BOARD for each TOPIC: HOW to MODERATE

What is PUBLIC HEALTH?

TYPES of EVIDENCE

Case studies

- TECHNICAL MODULES

Research Life Cycle

What's Next?

POSITIVE IMPACTS

IMPORTANCE in PUBLIC HEALTH RESEARCH!

WHY YOUNG PEOPLE?

What's in it for YOU?

DIVERSE FEEDBACK for RESEARCHERS

DID YOU KNOW? examples

BENEFITS:
- ✓ Communication
- ✓ Collaboration
- ✓ Leadership
- ✓ Confidence
- ✓ Advocacy

RIGHTS BASED

Paid / voluntary

DIVERSE FEEDBACK

HOW can YOUNG PEOPLE be INVOLVED? e.g. ROLES & RESPONSIBILITIES

SETTINGS/ DISCIPLINES: NGO's, UNIVERSITIES, HOSPITALS

Stages of Research /age etc.

ETHICS

Main concepts/ philosophies
- CONSENT & ASSENT
- POWER
- RESPECT
- TRANSPARENCY

ETHICAL RESEARCH VS. ETHICS in RESEARCH

POLLS: Before & After

- Sara's paper
- Reality shows
- Memes
- Ads

- Identify issues
- Collect data
- Share findings

HOW to SHARE/ IMPLEMENT LEARNINGS?

SET up GROUPS

ADVOCACY

Templates

Letters to council etc.

APPLY KNOWLEDGE to NEW CONTEXTS

VIDEO INTRODUCTION

- REFLECTION ACTIVITY
- ACTIVE LEARNING

LINK: Collective Action board, Research opportunities

HEALTH HIVE

TOGETHER WE THRIVE

MOOC Co-Creation Workshop

10TH MARCH 2025

CHARLES PERKINS CENTRE, THE UNIVERSITY of SYDNEY

GRAPHIC RECORDING by EMMA ROWLAND for TATUMKENNA.COM on GADIGAL LAND.

YOUTH ENGAGEMENT REFLECTIONS & Q/A

with DOMINIK → POSITIVE & VARIED experiences

WHY?

JARGON

✓ KEEP it SIMPLE
✓ PLAIN ENGLISH

OUTLINE

RESEARCH PROCESS

ACADEMIC WRITING SKILLS

HOW to PRESENT & TRANSLATE

DISSEMATION

"WHAT I would like to SEE in the MOOC"

CASE STUDIES

RETENTION RATES

LEARN about each OTHER

- ICE BREAKERS
- HOW to COMMUNICATE

EXPECTATIONS

- TIME ZONES etc
- TIMELINES

FACTORS that can IMPACT RESEARCH

Share OPPORTUNITIES

RESEARCH doesn't just lead to a PAPER

How the role could EVOLVE
- CONFERENCES etc

YOUR CONTRIBUTION MATTERS

REACHING other YOUNG PEOPLE

HEALTH HIVE
TOGETHER WE THRIVE

MOOC Co-Creation Workshop

10TH MARCH 2025 CHARLES PERKINS CENTRE, THE UNIVERSITY of SYDNEY

GRAPHIC RECORDING by EMMA ROWLAND for TATUMKENNA.COM on GADIGAL LAND.

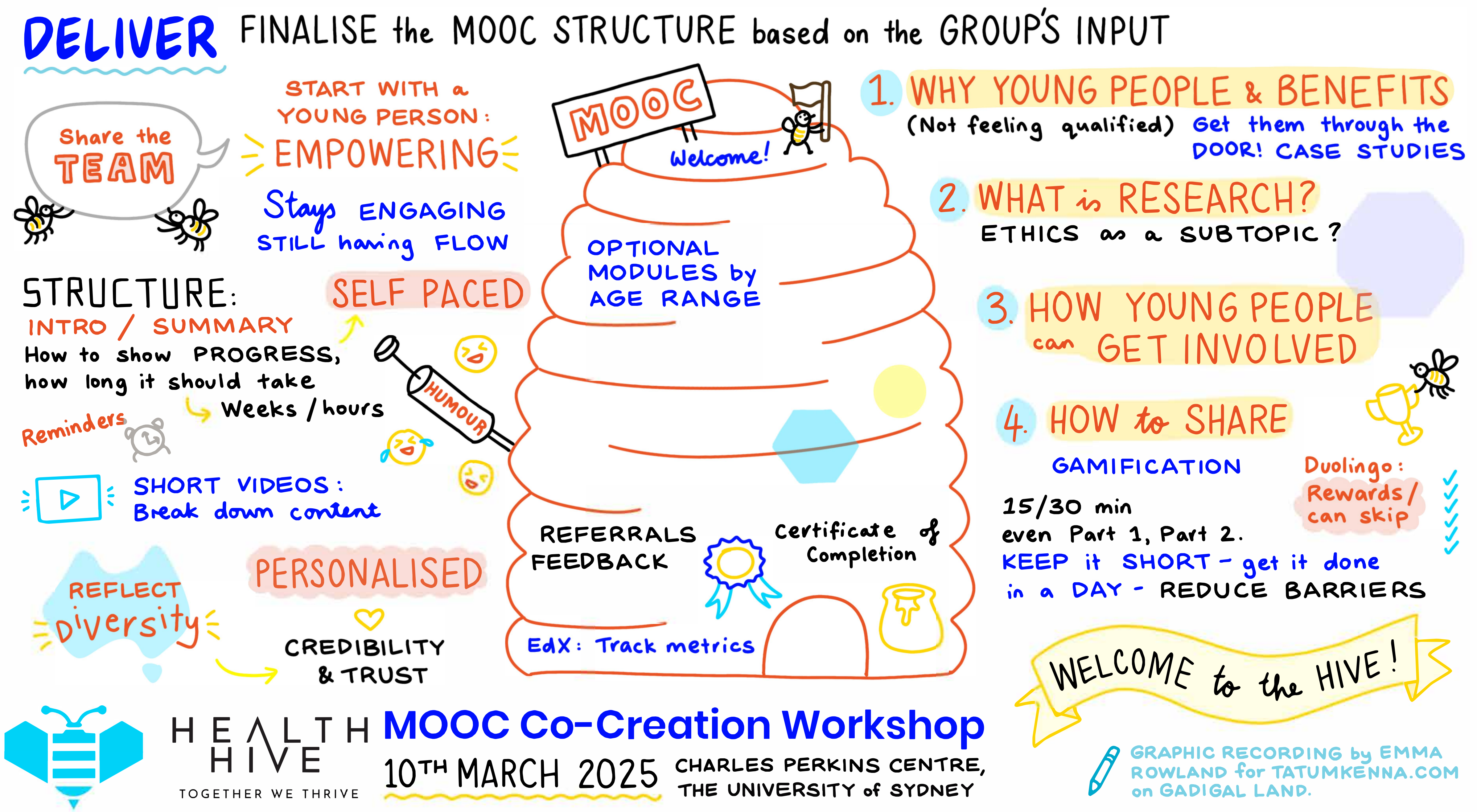
DELIVER
FINALISE the MOOC STRUCTURE based on the GROUP'S INPUT
Share the TEAM
START WITH a YOUNG PERSON:
EMPOWERING
Stays ENGAGING
STILL having FLOW
STRUCTURE:
INTRO / SUMMARY
How to show PROGRESS,
how long it should take
Weeks / hours
SELF PACED
Reminders
HUMOUR
SHORT VIDEOS:
Break down content
REFLECT Diversity
PERSONALISED
CREDIBILITY & TRUST
MOOC
Welcome!
OPTIONAL MODULES by AGE RANGE
REFERRALS
FEEDBACK
Certificate of Completion
EdX: Track metrics
1. WHY YOUNG PEOPLE & BENEFITS
(Not feeling qualified) Get them through the DOOR! CASE STUDIES
2. WHAT is RESEARCH?
ETHICS as a SUBTOPIC?
3. HOW YOUNG PEOPLE can GET INVOLVED
4. HOW to SHARE
GAMIFICATION
Duolingo: Rewards / can skip
15/30 min
even Part 1, Part 2.
KEEP it SHORT - get it done in a DAY - REDUCE BARRIERS
WELCOME to the HIVE!
HEALTH HIVE
TOGETHER WE THRIVE
MOOC Co-Creation Workshop
10TH MARCH 2025
CHARLES PERKINS CENTRE, THE UNIVERSITY of SYDNEY
GRAPHIC RECORDING by EMMA ROWLAND for TATUMKENNA.COM on GADIGAL LAND.
