# Supplementary material for: Building the Capacity of Adolescents as Researchers: The Co‐Creation of the Health Hive Online Course
Source: Health Expect. 2026 Jun 15;29(3):e70725. doi: 10.1111/hex.70725 (PMC13269657; doi:10.1111/hex.70725)
Supplement: Supplementary file 3 — Table S1: Checklist for reporting research with adolescent and youth engagement. [file HEX-29-e70725-s002.docx]

Table S1. Checklist for reporting research with adolescent and youth engagement

|  | **Item** | **Page number reported** |
| --- | --- | --- |
| Authorship | If applicable, list adolescents and youth as authors if they fulfil ICMJE authorship criteria; detail the criteria fulfilled | 1 |
| Acknowledgments | List adolescents and youth in acknowledgments if they did not fulfill ICMJE criteria. | Title page |
| Aim | If applicable, report the aim of adolescent and youth engagement in the study | 3-4 |
| Methods | | |
| Adolescents engaged | Provide a description of adolescents and youth involved with the engagement activity in the study | 1,4 |
| Stages of involvement | Report on how adolescents and youth were engaged at different stages of the study | 1, 4-6 |
| Level or nature of involvement | Report the level or nature of adolescent and youth engagement used at different stages of the study | 1, 4-7 |
| Discussion and conclusion | | |
| Overall influence | If applicable, comment on the extent to which adolescent and youth engagement influenced the study overall | 10 |
| Critical reflection | If applicable, comment critically on the study: reflect on things that went well and limitations of the process of adolescent and youth engagement (e.g., challenges, barriers) | 9,10 |

ICMJE=International Committee of Medical Journal Editors. Checklist adapted from Staniszewska et al (2017) by Nagata et al. (2025)
